# Seventh Annual Meeting of the American Society of Dental Surgeons

**Published:** 1846-09

**Authors:** 


### Miscellaneous Notices.

### SEVENTH ANNUAL MEETING OF THE AMERICAN SOCIETY OF DENTAL SURGEONS.

The Society met agreeably to adjournment on the 4th day of August, 1846, at 9, A. M., at the American Hotel, in the city of New York, and adjourned to the Hall of the Medical Department of New York University, in Broadway, opposite Bond street.

At 10, the Society convened at the last named place, and proceeded to business.

After the meeting was called to order, the minutes of the last meeting were read, and on motion, they were accepted and approved.

The President then called on the Recording Secretary for his report, in reference to the business transmitted to his hand, relative to the subject of amalgams. The report was then read, and is as follows, viz.

*Recording Secretary's Report.*

As the Recording Secretary of the American Society of Dental Surgeons, agreeably to a request made by this association, at their annual meeting held in the city of New York, in August, 1845, I issued the following circular, relative to the subject of amalgams.

This circular, which was forwarded to each member, on the 8th day of September, 1845, embodied the resolutions passed at the annual meeting, above referred to, and a *protest* against the use of amalgams, which the Society directed me to draft in accordance therewith; and I now report, that I have performed this duty imposed upon me by the Society, in accordance, as I trust, with the spirit which originated the measure, and I herewith transmit the result to the Society. This circular reads as follows, viz.

SYRACUSE, N. Y. *Sept.* 8, 1845.

DEAR SIR :

The American Society of Dental Surgeons have made it my duty to call your attention to the following Resolutions, unanimosly passed by this Association, at its annual meeting, held in the city of New York, August, 1845; and to request your compliance with the same.

AMOS WESTCOTT, *Recording Secretary.*

*Resolved,* That the American Society of Dental Surgeons, under the conviction that any *amalgam* whatever, whether used under the name of "Mineral Paste," "Adamantine Cement," "Succedaneum," "Diamond Cement," "Chinese Cement," "Lithodeum," "Alabaster Cement," or in any other way designated, is not only unfit, but dangerous when used for plugging the teeth, or their fangs—do hereby pronounce the use of amalgams, as malpractice.

*Resolved,* That the Recording Secretary be, and he is hereby directed, to forward to every member of this Society, within thirty days after the 10th day of August, 1845, a printed copy of the Resolutions passed at this meeting of the American Society of Dental Surgeons, relative to the use of amalgams under any form or name.

*Resolved,* That the Recording Secretary be requested, at the same time, to transmit to the several members as aforesaid, a printed protest, or certificate, in accordance with the said resolutions, notifying each member, that unless he shall subscribe with his own proper signature, the said protest or certificate, and return the same to the Recording Secretary, within sixty days from the time said notice is issued—or in cases of those residing or being out of the United States, within one year from the first day of August, 1845, he shall be considered as unworthy to be a member of the American Society of Dental Surgeons, and shall stand expelled from the same. And it is further

*Resolved,* That the Recording Secretary be and is hereby directed, without further reference, to strike from the roll of the Society, after the expiration of the above specified times, the names of such members as may have failed to comply with the terms and conditions as set forth in the foregoing resolutions.

I, ———, hereby certify it to be my opinion and firm conviction, that any amalgam whatever, whether used under the name of Mineral Paste, Adamantine Cement, Succedaneum, Diamond Cement, Chinese Cement, Lithodeum, Alabaster Cement, or in any other way designated, is not only unfit, but dangerous when used for plugging teeth or their fangs:

And I pledge myself never, under any circumstances, to make use of it in my practice as a dental surgeon:

And furthermore, as a member of the American Society of Dental Surgeons, I do subscribe and unite with them in their protest against the use of the same.

Given under my hand and seal, this —— day of ——, 184

{SEAL.}

Here follow the names of those who duly signed and returned the protest, within sixty days from the time it was issued, viz. * * *
* * * * * Total, 72.

Here follow the names of those who did not return the circular *within sixty days*, viz. * * * * * * Total, 62.

Of this last class, viz. those who failed to comply within the time prescribed by the Society, four, viz. * * * * returned their pledges after the time had expired together with the reasons of the delay, and petition the Society to consider them as having complied with the *spirit* of the resolutions.

Of those remaining, *two*, viz. * * have failed to comply in consequence of their not having received the circular. These also petition the Society to excuse them, on the ground that they were with the Society both in feeling and practice, and were prevented from complying with this requirement, by circumstances which they could not control. Since the sixty days, prescribed by the Society, expired, several have testified that they did not sign the *protest issued by* the *Recording Secretary*, in consequence of their having signed a similar protest which was drawn up *during the session* of 1845, and signed by most of the members present. Supposing that they had thus already met the requisitions of the Society upon this subject and that signing a second would be superfluous.

From facts which have come to my knowledge, I have good ground for believing that many others have failed to comply for similar reasons, while some have simply *neglected* to comply with said requirements.

Of those still remaining, three, viz. * * * in their response to the circular, *refuse* to comply with said requirements. Of these, one, viz. * declares himself friendly to the practice "in certain cases," and evidently bases his refusal on this ground. The other two, viz. * * declare themselves opposed to the *use* of all amalgams, but decline signing the protest, on the ground of their disapproving of the action of the Society, in passing the resolutions originating said protest. To present this subject in a more summary form, we may consider the whole number composing the Society, at the time the resolutions were passed, as divided into two general divisions, viz. the *first* embracing those who complied with the letter of the resolutions by signing and returning the protest within sixty days.

| | |
|---|---|
| The whole number of these is | 72 |

*Second*, embraces those who failed to comply with the letter of said resolutions.

| | |
|---|---|
| The whole number of these is | 62 |
| Total | 134 |

The second division may be divided into *four classes*, viz.

1st. Embracing those who signed and returned the protest after the sixty days had expired.

| | |
|---|---|
| Of these there are | 4 |

2d. Embracing those who report that they did not receive the circular.

| | |
|---|---|
| Of these there are | 2 |

3d. Embraces those who did not respond to the circular by letters addressed to me as Recording Secretary. These are, in all 53

4th. Embraces those who *refuse* to sign the protest.

Of these there are 3

It appears, therefore, that, of the whole number, *seventy-eight* have either signed the protest issued by me, or have expressed their entire concurrence in the sentiments embodied in the resolutions directing its issue—that *fifty-three* have not, for sundry reasons, *directly* responded to the circular—and that *one* only has in his response, declared himself friendly to the practice of using amalgams for dental purposes, and that only in "certain cases."

Now, the resolutions accompanying the protest direct the Recording Secretary to strike from the roll of members the names of those who have failed to comply with the requirements within sixty days—or in case of those living or being out of the country, in one year. But a question has arisen in my mind, as to whether in the discharge of this duty, I was to consult the spirit or the letter of these resolutions? If the former, they certainly could not make it obligatory upon me, to strike from the roll of members, the names of those who had given undoubted evidence that they were wholly opposed to the use of amalgams.

This would indeed be *counter* to the feeling and spirit which originated these resolutions. The Society had but a single object in passing them, and this was not to expel members opposed to the practice proscribed thereby, but have them serve simply as a means to detect and expel, those who were actually engaged in the practice in question.

I did not, therefore, feel in duty bound to strike from the roll of members, the names of those who *have*, although subsequent to the expiration of the *prescribed* time, returned the protest duly signed—nor yet the names of those who have shown that they were only prevented from doing so by circumstances which they could not control. I have already stated that of those who had not in the prescribed form, responded to the circular, I had good reasons for believing that many were with the Society, both as regards feeling and practice, respecting the use of amalgams—nor is it my prerogative on this occasion to declare, that *any* of these are friendly to or are engaged in this practice. Viewing the subject in this light, I beg leave, therefore, to report that I have not stricken from the roll of members, the names of those embraced in either of the following classes, viz.

1st. Those who signed and returned the protest, although after the expiration of the time prescribed for them to do so—or

2d. Those who have given satisfactory reasons for their delinquency—or

3d. Those who have not responded to the circular.

In regard to those embraced in the first two of the above named classes, there can be no doubt of the *impropriety* of striking their names from the roll of members. And in respect to those embraced in the last class, the mere fact that they did not respond to the circular, is as good evidence, (unless some extrinsic evidence proves it otherwise,) that they did not receive the circular, as that they are friendly to the practice against which the protest was made. It is a fact clearly exhibited by a simple presentation of the names included in this class, that a large majority of those embraced in it are wholly opposed to the use of all amalgams for dental purposes. It, indeed, embraces several who not only advocated, but voted for the resolutions originating the protest and prescribed its form.

I, therefore, respectfully beg leave to submit this question again to the Society, not feeling it necessarily incumbent upon me to strike these names from the roll without further orders from the Society, made with reference to the foregoing facts and considerations.

A. WESTCOTT, *Recording Secretary.*

This report was accepted.

Some debate here arose as to whether "striking the names from the roll," was necessary in order that the expulsion anticipated in the resolutions, should have taken place, and the report was at length referred to a special committee.

A committee was also appointed to arrange the business of the meeting.

Adjourned to 4, P. M.

Met at 4, P. M.

Dr. Bridges read his address: a copy was requested for publication.

An essay was then read from Dr. Elliott, by Dr. C. A. Harris, entitled "Contributions to Mechanical Dentistry, No. 9:" ordered to be published.

Adjourned to Wednesday, 9, A. M.

*Wednesday*, 9, A. M.

The first business was to hear the report of the committee to whom was referred the Recording Secretary's report.

The report of this committee not being adopted, the Secretary was ordered to make no record of it, except to refer to it. The subject of the Recording Secretary's report was again taken up as left before it was referred to a committee, and it was decided that the expulsion, if it could take place at all under the resolutions, must have been effected by virtue of the *third* resolution of the circular, and hence the direction contained in the fourth, to strike certain names from the roll, was not necessary to consummate such expulsion.

The President, therefore, gave notice to those present who had failed to comply with the requirements of the resolutions originating the circular, that they could not be regarded as members till some provision was made by which they could be restored.

The remainder of the day was occupied in devising some plan by which the Society could at once be protected against this practice, and at the same time do justice to many who would innocently suffer by the unmitigated action of the resolutions.

Adjourned to Thursday, 9, A. M.

Met Thursday, 9, A. M., pursuant to adjournment.

The minutes of the meeting were read to the present time and approved.

The committee to whom was referred the business of amending and revising the constitution and by-laws, presented a very able and labored report, materially changing many important features, both in the constitution and by-laws. The most important of these pertained to the admission of members—throwing around the Society far stronger guards than exist in the constitution now in operation relative to this particular. This report required that candidates should, under all circumstances, be examined either by the regular examining committee, or by some person or persons deputed by them to perform this office.

This report was accepted, and a vote of thanks tendered the committee for the great labor bestowed upon it.

Wm. H. Dwinelle moved that the constitution and by-laws, as amended by the committee in said report, be adopted as a whole.

Before the vote was taken, Dr. C. A. Harris offered the following amendment, viz.

"Any graduate of a regularly chartered dental college, who shall have been in practice three years, shall not be subject to an examination before the examining committee of this Society."

The voting upon this amendment resulted in a tie, and the President declining to give the casting vote, it was lost.

The original motion by Dr. Dwinelle was withdrawn, that the Society might proceed to other business.

Adjourned to 4, P. M.

Met at 4, P. M., pursuant to adjournment.

The following gentlemen were proposed as candidates for election as *honorary members*, viz.

1. Dr. James Robertson, of London, presented by Dr. C. A. Harris.
2. Dr. John Martin, of Portsmouth, England, presented by E. Parmly.

Both of the above were unanimously elected.

Letters of *resignation* were read from the following members, viz. John C. McCabe, C. F. Martin and Moses Holmes. Accepted.

The subject of the report of the committee on constitution and by-laws was again taken up, and, with a view finally to dispose of this report for this session, it was moved by A. Westcott, that the report of the committee be adopted as a whole. Lost.

A letter was read from Dr. Solyman Brown, tendering his resignation.

Dr. C. A. Harris moved that Dr. Brown's resignation be not accepted. His letter was referred to a committee, with directions to report at the next annual meeting.

This committee was appointed by the *chair*, and is as follows: C. A. Harris, E. Noyes, J. H. Foster.

Adjourned to 9, to-morrow.

*Friday*, 9, A. M.

On motion of Dr. J. B. Rich, the following amendments to the constitution and by-laws were unanimously adopted, viz.

To amend the first section of the third article of the constitution by striking out all after the word "executive," in the third line, and substituting the following: "committee consisting of seven members, and a publishing committee consisting of three members."

To amend the fourth article, by striking out the first section, and substituting the following:

"Section 1. There shall be two classes of members, acting members and honorary members. The first class shall consist of those who have been duly elected as active members of this Society, and who shall have signed and adopted this constitution, and have paid into the treasury the initiation fee and dues required by the laws, and have otherwise complied with the requirements of the by-laws. The second class shall consist of those who have been duly elected as honorary members of this Society."

To amend the same article, by striking out all after the figure 2, in the first line of the second section, and substituting the following:

"All applicants for membership to this Society shall be at least twenty-one years of age, shall have a good English education, shall produce evidence of unexceptionable moral character; and shall have studied and practised for the full term of two years, in a regularly chartered Dental College in any of the United States, or with some respectable dental practitioner, known as such to this Society; and have practised in addition, as a dental surgeon, for the full end and term of three years subsequent to the above-mentioned term of instruction. Or, in lieu of the aforesaid five years of studentship and practice, he shall have practised as a dental surgeon for the full term of six years."

To amend the same article, by adding the following as the fifth section:

"Section 5. When a member of this Society desires to withdraw his membership, he shall signify his desire in writing, accompanied by his certificate of membership or diploma, and if he is clear of the books, and no charges be preferred against him, he shall be entitled to withdraw; his certificate of membership or diploma shall be cancelled, and he shall no longer be considered as a member of this body."

To amend the fifth article, by placing the word "section" and the figure "1," at the commencement of the first line, and striking out all after the word "all" in the first line, and substituting the following:

"Applications for membership, as acting members of this Society shall be in writing, and recommended by at least three members, who are personally acquainted with the professional qualifications and moral character of the applicant, and are willing to vouch for the same; they shall be made to the secretary of the executive committee, at least six months previous to a regular annual session. All propositions for the election of persons as *honorary* members, shall be signed by at least three members of this Society, who are willing to vouch for the professional qualifications of the person so proposed, and shall be addressed to the secretary of the executive committee. The said committee shall, if possible, investigate the subject immediately, and report to the Society as soon as practicable; and the candidate may be ballotted for at any regular annual session as soon as the committee report."

"Section 2. No application for active membership in this Society shall be considered until the full term of six months after the application shall have been received by the executive committee, in the manner described in the first section of this article, and at no other than a regular annual session. Propositions for membership as honorary members may be considered as soon as the report of the executive committee upon such proposition has been received by the Society, which cannot be acted upon except at a regular annual session."

"Section 3. The executive committee shall present to the Society, at the regular annual session, the names of all persons who have made application for admission in regular form, accompanied by a report of the result of their investigations, in each individual case."

"Section 4. All candidates for membership shall be ballotted for, with ball ballots; and if there should not be one black ball for every six acting members present, the even sixes only counting, the candidate shall be duly elected. But if there should appear against him one black ball for every six acting members present, the even sixes only counting, he shall be rejected, and so declared."

"Section 5. Every applicant, on being admitted to membership, shall pay into the treasury the sum of twenty-five dollars, as an initiation fee. He shall also pay, as annual dues, the sum of two dollars and fifty cents, at every annual session thereafter."

"Section 6. Every member of this Society in good standing, who shall be admitted after the passage of this section, shall be entitled to the diploma of the society without fee. Those who may have been in membership previous to the passage of this section, and who remain in good standing, shall be entitled to the diploma upon the payment of five dollars."

To amend the sixth article, by striking out all after the word "Any" in the first line, and substituting the following: "member or members of this Society may be expelled from this body for immoral conduct, malpractice in business as declared to be such by this Society, or for refusing to comply with the requirements of this constitution and by-laws, or the requirements of this Society, or other sufficient cause, on the motion of one member seconded by another, at any regular annual session, in which case a majority of two-thirds of the members present shall be required."

To amend the eleventh article, by striking out the whole of the first section, it having been embodied in the fourth article.

And to strike out the figure "2" after the word "section," in the first line of the second section, and substituting in its place the figure "1."

To amend the first section of the tenth article, by striking out all after the words "three-fourths" in the third line, and substituting the following, "of the acting members present shall be required to effect the act of dissolution."

To amend the fifth section of the first article of the by-laws, by striking out all after the word "secretary" in the first line, and substituting the following, "to enter in a book kept for that purpose an accurate record of all the proceedings of the Society during his term of office, and to furnish copies of any part or parts thereof, at the order of the president or his substitutes. He shall also keep the seal of the Society, together with the plates from which diplomas, certificates or degrees are struck; also all the records belonging to the Society, designed to be preserved; and he shall, furthermore, countersign, and set the seal of the Society to all diplomas and other instruments requiring the same, he shall at least, three months previous to the regular annual session, notify every member of the time and place of meeting, and of the names, residence, and reference of all those persons who have applied for membership as acting members of this Society, and whose cases will be acted upon at the said regular annual session. He shall also deliver all books, papers, seals, plates and other articles appertaining to his department, to his successor in office, or to a committee of the Society, duly authorised to receive them."

To amend the ninth section of the first article of the by-laws, by striking out all after the figure "9," in the first line, and substituting the following:

"It shall be the duty of the executive committee to audit the accounts of the Treasurer, and report on the same to the Society at its annual sessions; to arrange and lay down the order in which the business of the Society shall be transacted at its sessions. And it shall also be the especial duty of such committee to receive all applications for membership that may be presented to them in regular form, according to the requirements of the constitution, and accompanied by the proper vouchers, and to reject those applications which may come to them in irregular form. They shall diligently inquire into the professional reputation of all candidates, who may be proposed as honorary members, and ascertain whether they are entitled to such distinction. They shall in the case of all applicants for membership, as acting members of this Society, submit them to an actual and thorough examination, in all the departments of dental surgery, except in the case of those who are exempted from such examination by the first section of the eleventh article of the constitution; and they shall take every means in their power to understand the general standing and moral character of the said applicants, they shall, after such examination, present the names of the applicants to the Society, as candidates for membership, accompanied with a written report of the result of their investigations, and of all the facts which may have come to their knowledge, relating to each individual case. They shall organise immediately after their election, and elect from their own body, a chairman and secretary. The duty of the chairman shall be to preside at the meetings of the committee.

"The duty of the secretary of this committee shall be to receive all applications for membership, and to lay the same before the committee, immediately on his reception of such applications; he shall be both the recording and corresponding officer of the committee, and he shall also enter in a book kept for that purpose, an accurate record of all the transactions of the said committee, and of the examinations conducted by them, together with all the facts which may come to their knowledge, relating to candidates for membership, who may make application during his term of office, and it shall be his duty to write, and sign all the reports emanating from the committee, and have the minutes written out, so that the committee may present the record if it should be needed, at the time the names of the candidates are presented to the Society for its action, and he shall furnish copies of any part or parts thereof, at the order of the President or his substitutes, and also to deliver the books and proceedings, together with all the books and papers appertaining to his department, to his successor in office, or to a committee duly authorised to receive them."

To amend the second article of the by-laws by striking out the word "and" after the word "recording," in the second line, and adding after the termination of the word "secretaries," in the third line, the words and "treasurer."

This morning was principally occupied in discussing measures for the restoration of those who were reported as delinquents in regard to the requisition of the Society pertaining to the use of amalgams, and who were desirous of retaining membership, on the general terms of those resolutions. In pursuance of this object, it was on motion of A. Westcott—

*Resolved,* That all the action previously taken at *this* session relative to the disposition of those members who had failed to comply with the requirements of this Society, made at its annual meeting in August, 1845, relative to the subject of amalgams, be *reconsidered.* Unanimously carried.

On motion of C. A. Harris—

*Resolved,* That all the resolutions and action, alluded to in the above resolution be, and they are hereby rescinded. Unanimously adopted.

Whereupon the following preamble and resolutions were offered by John B. Rich, and unanimously adopted:

Whereas, the report of the recording secretary, relative to the subject of amalgams, shows that several of the members did not receive the circular which the Society directed him to issue, seasonably to comply with the requirements thereof: and—

Whereas, said members did finally comply with said requirements, by signing and returning said protest; and, whereas, certain others have certified that they did not receive said protest at all, but that they were fully prepared to concur in its sentiments, and they would have complied with the requirements of the resolutions, had the circular reached them:

And, Whereas, said report of the recording secretary shows that a large number of the members have failed to respond to said circular, either by *refusing* to comply with the resolutions which accompanied it, or by *signing* said protest.

And, Whereas, this report furnishes evidence that some of these did not receive this circular, and, whereas, said report furnishes *no proof* that any so neglecting to respond to the said circular failed to do so, because they were either friendly to, or were engaged in the said practice of using amalgams for dental purposes.

***Resolved,*** That while we retract nothing as regards our sentiments expressed in these resolutions, relating to the use of amalgams, or our belief, that the Society have full powers to enforce them, and while we are still determined to free ourselves from the odium necessarily attached to this reprehensible practice, nevertheless, in the opinion of the Society, these resolutions have not in their ***practical operations*** secured equal and exact justice, to all of the members of this association: and it is therefore ***resolved,*** that the second and third resolutions which accompanied the protest be and they hereby are ***repealed.***

***And it is further resolved,*** That in the opinion of this Society, those who did actually sign and return the protest, although after the expiration of the sixty days, and those who have expressed to the recording secretary an entire concurrence in the sentiments of said protest, have complied with the spirit of the resolutions. And that their cases shall not again be acted upon by virtue of these resolutions.

***And it is further resolved,*** That those members whose names appear in and constitute the third and fourth classes of the recording secretary's report, be, and hereby are *required* to sign and return to the said secretary, the following protest:

I, ———, hereby certify it to be my opinion and firm conviction, that any amalgam whatever, whether used under the name of Mineral Paste, Adamantine Cement, Succedaneum, Diamond Cement, Chinese Cement, Lithodeon, Alabaster Cement, or in any other way designated, is unfit for plugging teeth or their fangs:

And I pledge myself never, under any circumstances, to make use of it in my practice as a dental surgeon:

And furthermore, as a member of the American Society of Dental Surgeons, I do subscribe and unite myself with them in their protest against the use of the same.

Given under my hand and seal, this —— day of ——, 184 at ——, in the state of ——.

{SEAL.}

*It is further resolved,* That the recording secretary be directed to forward to each member embraced in the third and fourth classes of the secretary's report, a copy of the original resolutions, together with a copy of the foregoing preamble and resolutions; and also the above mentioned protest, respectfully requesting that each member so addressed shall promptly respond to this communication, either by signing the protest accompanying it, or by *declining* to do so, in order that the secretary may be enabled to make a definite report to the Society, in respect to each individual case, and that the Society may be enabled to act more advisedly upon the case of each one who does not signify his desire to withdraw from the Society previous to the next regular annual session.

*Resolved,* That the recording secretary be directed to transmit to the Society, at the annual session of 1847, an accurate report of the result, that the case of each may be duly acted upon by the Society, at the last named annual meeting, in accordance with the testimony *thus* furnished.

Dr. C. O. Cone offered some very appropriate remarks relative to the adoption of some system that should be uniform among the members of the Society, in regard to transmitting useful facts and information to each other, and to the profession. At the close of his remarks, he offered the following resolutions which were adopted, viz.

Whereas, it being an admitted truth, that experience, aided by observation and comparison, has been the main object in establishing facts which has rendered the utility of dental surgery such as to deserve the gratitude of mankind, and elevate it to the dignity of a respectable, learned and responsible profession, and it being one of the avowed objects of this Society to render such facilities as it shall be enabled to, to multiply such facts, "the advancement of the science (dental surgery) by free communication, and interchange of sentiment," and believing that the object of the Society upon this subject will be more fully carried out by some systematic course.

*Resolved,* That this Society appoint a committee to prepare and report to the Society, a tabular sheet, to be used by its members to make an annual report to the Society, of all their operations, and the characteristics of the teeth and mouth, upon which such operations are performed, together with a description of the treatment and preparation of the mouth for, and the application of all artificial pieces, together with a description of all previous failures, in either surgical or mechanical dentistry, that may come under their treatment, together with what, in their judgment they considered the cause of such failures.

*And be it further resolved,* That it be the duty of each member of the Society, to make an annual return to the Society of such report of their practice as is above mentioned at its annual meeting: *and be it further resolved,* That the Society appoint a committee to condense such statistics, and publish the same with the doings of the Society, for the benefit of its members.

The committee appointed by the Society to carry out said resolutions, were the following, viz.

C. O. Cone, *Chairman*, E. Parmly, C. C. Allen, J. H. Foster, E. J. Dunning.

The executive and examining committee have examined the Treasurer's books, and found the following to be the result:

| | |
|---|---|
| There has been received for Journal and dues, . . . . . . . | $ 1483 24 |
| There has been expended, . . . . . . . . . . . . . . . . . . | 1433 62 |
| There is due the Journal, about . . . . . . . . . . . . . | 1700 00 |
| Now due for annual dues, about . . . . . . . . . . . . | 900 00 |
| In the hands of agents in this country that may be had at any time, about . . . . . . . . . . . . . . . . . . . . | 180 00 |

On motion of A. Westcott, a committee was appointed to prepare *a code of ethics* by which the Society might be regulated in many particulars not specified in the constitution and by-laws.

This committee are—A. Westcott, E. Parmly, C. C. Allen.

On motion of C. C. Allen—

*Resolved,* That the essays on dentistry printed some time since by this Society, be distributed gratuitously among all its members who shall not be in arrears, at the close of the present meeting for the annual dues. A list of the above members to be furnished to Mr. A. W. Brown, who now has charge of them.

The executive and examining committee reported that they had examined the report presented by the Treasurer in conjunction with the books and vouchers of his department, and find the said report to be correct.

On motion of C. A. Harris, the constitution and by-laws presented by the committee on constitution and by-laws was referred back to said committee, with instructions to report at the next annual session.

On motion—*Resolved,* That the editors receive twenty-five dollars in addition to the compensation received last year.

Adjourned to 8 o'clock, P. M. to meet at the house of Dr. C. C. Allen.

*Friday,* 8 P. M.

The Society met agreeably to adjournment at Dr. C. C. Allen's.

J. W. Smith tendered his resignation—accepted.

The Society next proceeded to the election of members. The names of several candidates were in the hands of the executive committee, but could not be acted upon as the amendments of the constitution and by-laws adopted at this session required that each applicant should be notified and have the privilege of withdrawing his name in case the conditions were not satisfactory. The result was, that one only, viz. Dr. A. Van Camp, of Nashville, Tennessee, was elected.

On motion, it was *Resolved,* That the committee on constitution and by-laws, be directed to publish 200 copies of the constitution and by-laws, as amended, for distribution among the members.

The committee on the case of J. Smith Dodge, reported progress.

Dr. J. C. Ross, of Lexington, Kentucky, was excused for non-attendance.

The Society next proceeded to the election of officers, which resulted as follows, viz.

| | |
|---|---|
| E. PARMLY, | PRESIDENT. |
| L. ROPER, | 1ST VICE-PRESIDENT. |
| E. NOYES, | 2D VICE-PRESIDENT. |
| JOHN ALLEN, | 3D VICE-PRESIDENT. |
| C. A. HARRIS, | CORRESPONDING SECRETARY. |
| A. WESTCOTT, | RECORDING SECRETARY. |
| C. A. HARRIS, | TREASURER. |
| J. H. FOSTER, | LIBRARIAN. |

*Editors of the American Journal and Library of Dental Science.*

C. A. HARRIS, A. WESTCOTT, E. J. DUNNING.

The committees and essayists of last year were all to stand for the present year, except that W. H. Dwinelle stands as a substitute for J. B. Rich, who declined serving.

Adjourned to meet at *Saratoga Springs*, on the first Tuesday in August, 1847. A. WESTCOTT, *Recording Secretary.*

